# A preliminary assessment of mercury, methylmercury and other potentially toxic elements in largemouth bass (*Micropterus salmoides*) from the Almadén mining district

**DOI:** 10.1007/s10653-024-02326-3

**Published:** 2024-12-23

**Authors:** J. I. Barquero, J. J. Hidalgo, J. M. Esbrí, P. Higueras, E. García-Ordiales

**Affiliations:** 1 Instituto de Geología Aplicada, Universidad de Castilla-La Mancha, Pl. Manuel Meca 1, 13400 Almadén, Ciudad Real Spain; 2 Escuela de Ingeniería Minera e Industrial de Almadén, Pl. Manuel Meca 1, 13400 Almadén, Ciudad Real Spain; 3 Facultad de Ciencias Químicas, Universidad de Castilla-La Mancha. Av, Camilo José Cela 1, 13071 Ciudad Real, Spain; 4 Departamento de Mineralogía y Petrología, Universidad Complutense de Madrid, C. José Antonio Novais 12, 28040 Madrid, Spain; 5 Departamento de Explotación y Prospección Minera, Escuela de Ingeniería de Minas, Energía y Materiales, Universidad de Oviedo, Independencia, 13, 33004 Oviedo, Spain

**Keywords:** Mercury, Almadén, Selenium, Food risks, *Micropterus salmoides*

## Abstract

The food chain of the Valdezogues River system is at considerable risk due to the presence of mercury in the environment and to intense bioaccumulation and biomagnification processes in some fish species, particularly in piscivorous. Moreover, the presence of mercury in fish is a reliable indicator of the presence of its most toxic form, methylmercury. Of interest is that selenium, when present together with mercury in food, represents a significant decrease in the risks related to the ingestion of methylmercury. This study presents the concentrations of total mercury, methylmercury, selenium, and other elements in *Micropterus salmoides* from a transect of the Valdeazogues River. This water course cuts across the Almadén mining district, which has been the most important producer of this element for over 2000 years. Results highlight the highest total- and methylmercury concentrations ranging between 0.47 and 7.61 mg kg^−1^ ww for total mercury, and with methylmercury representing between 79.7 and 92.1% of total mercury. These are the highest concentrations in the element ever found in this species. Seven sampling sites were examined along a 34 km long transect of the river, starting at an open pit lake corresponding to a decommissioned Hg mine (El Entredicho open pit, isolated from the river course), with the rest of the sites located downstream. Concentrations of mercury are the highest in this open pit lake and decrease steadily downstream. Concentrations of selenium are also high to very high, ranging between < 2.5 and 11.4 mg kg^−1^ ww. The Se/Hg molar ratio, as well as the HBV_Se_ index, show values indicating low risk, except for specimens of low size/age from the most highly Hg contaminated site considered in this study. The rest of the potentially toxic elements (Se, As, Pb, Zn, Sb and Cu) show no concerning values even though the area is heavily populated with decommissioned polymetallic mines.

**Supplementary Information:**

The online version contains supplementary material available at 10.1007/s10653-024-02326-3.

## Introduction

**Fig. 1:**
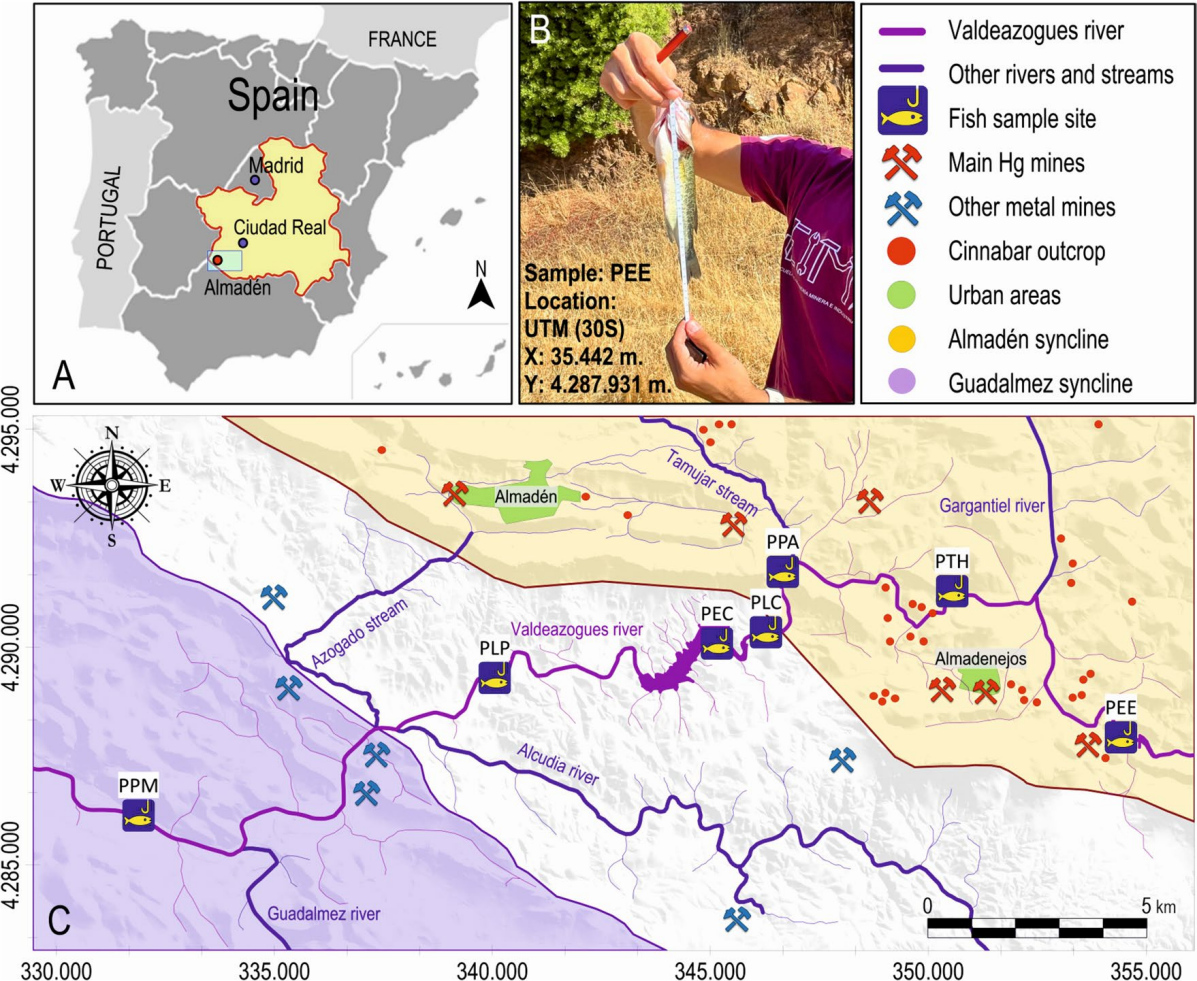
Location of the sampling area (**A**); one of the captured exemplars (**B**); and location of the fishing sites and of the most prominent Hg and other PTE mines (**C**)

The Almadén mercury mining district is located in the southwest of the Ciudad Real province, in the Castilla-La Mancha region, south central Spain (Fig. 1). It is well known for the presence of several cinnabar (HgS) mines (Hernández et al., 1999; Saupé, 1990, among others), active for more than 2000 years. The district comprises one vast mine, having produced more than 95% of the total production of the district, and almost one third of the total world production of this element. Additionally, there are four smaller mines, as well as more than 50 sites where the presence of cinnabar has been documented (see Hernández et al., 1999 for more details). All these sites are spread over an area of some 125 km^2^ and are all comprised in a single Variscan tectonic structure: the Almadén syncline, integrated in the southernmost Central Iberian zone of the Iberian Massif (García Sansegundo et al., 1987). To the south of this structure, it extends the Alcudia anticline and valley, which has been the site for base metal mining, particularly Pb–Zn-Ag, with lower concentrations of As, Cd and Sb (Palero et al., 2003). More to the south, the Guadalmez syncline (Lorenzo et al., 2005) is mostly characterised by the presence of minor Sb deposits (Barquero et al., 2022; Gumiel & Arribas, 1987). As shown in Fig. 1, the Valdeazogues River also transects these two major geological structures, and receive inputs containing wastes from the mentioned Pb–Zn–Ag and Sb decommissioned mines.

The drainage system of this area is characterised by semistational rivers, tributaries of the Guadiana River. As shown in Fig. 1, the most important watercourse is the Valdeazogues River, running through part of the Almadén syncline (passing in this segment through the El Entredicho mining site), and part of the Alcudia Valley, where at this segment of the river waters from its tributaries are received, namely the Gargantiel River draining the area where the Las Cuevas mine is located, the Tamujar stream, which contains no significant Hg pollution sources; and the Azogado stream, draining the extremely highly Hg-polluted area of the Almadén mine area. Different authors such as Berzas-Nevado et al. (), Gray et al. (2004), García-Ordiales et al., (2014, 2016a, 2016b and 2016c, 2017 and 2018) have studied the presence of total Hg (THg) and methylmercury (MeHg) in the waters and sediments of the Valdeazogues River basin, as well as in some of its aquatic fauna, including freshwater bivalves (Berzas-Nevado et al., 2003) and crayfish (Higueras et al., 2006). These studies have revealed concentrations of THg and MeHg much higher than those recommended for food usage of these organisms. For instance, the study by Berzas Nevado et al. (2003) on the Valdeazogues River ecosystem showed the presence of Hg in bivalves (*Unio pictorum*) between 0.7 and 1.0 µg g^−1^ (wet weight), of which 20–40% was methyl-Hg ([CH_3_Hg]^+^). These concentrations exceed the maximum allowable concentration according to the USEPA (2001) [CH_3_Hg]^+^ criterion of 0.3 µg g^−1^ (wet weight) for crustacean tissue. Besides, THg concentrations in crayfish (*Procambarus clarkii*), frequently consumed by the local population have been recorded at over 6.5 µg g^−1^ (wet weight) in muscle tissue (Higueras et al., 2006). These values by far exceed the limits recommended by the European Commission (2006) and the U.S. Food and Drug Administration (2020) for THg in bivalves and crustaceans (crayfish), set at 0.5 µg g^−1^ and 1.0 µg g^−1^, respectively. Moreover, García-Ordiales et al., (2014, 2016a, 2016b, 2016c) highlighted the presence of As, Co, Hg, Pb, and S showing significant enrichment and contamination in the bottom sediments of the fluvial system centred in the Valdeazogues River.

Largemouth bass (*Micropterus salmoides*) is a freshwater fish introduced into central Spanish rivers during the twentieth century as a species for sport fishing. It is a predatory species, and is located at the top of the regional fluvial food chain; it is particularly vital to note that the main targets of their predatory activity are crayfish, which are abundant in the basin, and likely the previously mentioned freshwater bivalves, both species with proven high concentrations of Hg and MeHg in this area (Berzas-Nevado et al., 2003; Higueras et al., 2006). A considerable number of published studies analyse this species for Hg and MeHg all over the world (107 references were found on Scopus.com using the search terms "mercury" and "*Micropterus salmoides*", in the period between 1972 to present day, reflecting the extensive availability of studies and data regarding mercury presence in this species over several decades).

Mercury is a well-known toxin, and MeHg ([CH_3_Hg]^+^) is an organomercurial species with extreme toxicity, in particular for aquatic environments as it bioaccumulates in fish. This caused the catastrophic poisoning of thousands of inhabitants of the Minamata Bay area in southwest Japan due to the massive release of this compound into the bay by a chemical factory (Harada, 1995; Kurland et al., 1960). Since then, many published studies have assessed the bioaccumulation of Hg and MeHg in fish (Mason et al., 1995; Westöö, 1966, among many others), and a great number of these studies highlight the effects of this bioaccumulation on human health (Clarkson & Magos, 2006; Mozaffarian & Rimm, 2006). Such studies conclude that dietary MeHg bioaccumulates in fatty fish in particular, and more than 75% (and up to 99%) of the total Hg present in this kind of fish species corresponds to MeHg (Fitzgerald & Clarkson, 1991; Bloom, 1992; Lescord et al., 2018).

Selenium (Se) is an essential trace element needed by most organisms within a physiologically appropriate margin and which is toxic at high levels. This element exists in the biota in the form of selenoproteins such as selenocysteine and selenomethionine and is incorporated into the active sites of antioxidant selenoenzymes (Steinbrenner & Sies, 2009; Taylor et al., 2009; Ralston & Raymond, 2010, 2018; Raymond & Ralston, 2020). However, Se can also be harmful to the biota at high doses due to the narrow margins between the levels associated with deficiency or toxicity (Wang & Gao, 2001; Taylor et al., 2009; Sharma et al., 2017). In recent years, the scientific community has focused on the important relationships between Se and Hg in term of the benefits/risks to the environment: a considerable number of scientific studies have confirmed that Se–Hg antagonism is a widespread phenomenon in microorganisms, fish, poultry, humans, and other mammals (Ganther et al., 1972; Koeman et al., 1973; Prohaska & Ganther, 1977; Skerfving, 1978; Cuvin-Aralar & Furness, 1990, 1991; Raymond et al., 2012; Falnoga & Tušek-Ẑnidarič, 2007; Yang et al., 2008; Khan & Wang, 2009; Gajdosechova et al., 2018), and suggesting that a universal Se–Hg antagonism may exist among various species (Økelsrud et al., 2016; Ralston & Raymond, 2018; Sanz-Prada et al., 2022).

On these bases, this study presents and discusses the extremely high concentrations of Hg and MeHg, as well as other potentially toxic elements (PTEs), in the muscle of largemouth bass (*Micropterus salmoides*) from a transect of the Valdeazogues River, including the isolated waters present in the open pit lake of the decommissioned El Entredicho Hg mine. Particular emphasis was placed on the relationship between Hg and Se contents in the analysed specimens, aimed at clarifying the possible risks related to the occasional consumption of this fish species, although this is a hobby in decline, going from some 23,000 to 8,000 fishing licenses in Ciudad Real province in the last 20 years.

### Background: total- and methylmercury concentrations in water and sediments

The following summary contains previously published information regarding the presence, abundance, and distribution of Hg and other PTEs in the water and sediments from this district. The survey done by Gray et al. (2004) provides the earliest data for total MeHg and Hg (THg) concentrations in water and sediments from the Almadén district, corresponding most closely to the Valdeazogues River, ranging between 3.0–2300 ng L^−1^ (MeHg) and 0.32–82.00 mg kg^−1^ (HgT). Moreover, García-Ordiales (2014) provides data on the concentration of particulate-bound Hg (corresponding to water filtered < 0.45 µm). According to this research, 80% of the water samples contained dissolved Hg (DHg) in the range between 10 and 50% referred to THg. Also, García-Ordiales et al. (2014) described another survey regarding sampling and analysing stream sediments in the area, including the Valdeazogues River and the Azogado stream. They analyse not only Hg, but also major and minor elements, including the PTEs Pb, Zn, Cu, As, among others. Pb ranges between 27 and 131 mg kg^−1^, with the lowest values in tributary from the high course of the basin, and maximum values obtained to the south of Almadén, where some now disused Pb-Ag mines are located; Zn ranges between 65 and 320 mg kg^−1^, with a similar distribution of higher and lower values as that of Pb; Cu ranges between 12 and 50 mg kg^−1^, with low variability among the different sites and with no coincidence of maximum and minimum values of Pb and Zn; As ranges between 9 and 17 mg kg^−1^, with a notably random distribution of similar values. Finally, Hg ranges between 6 and 256 ng g^−1^, with minimum in the sediments of the uncontaminated Alcudia River, low values in the Tamujar tributary, in the higher course of the Valdeazogues River and in the Castilseras reservoir and downstream the reservoir. Values over 100 ng Hg g^−1^ are present in the rest of the basin, including values > 200 ng g^−1^ in the highly contaminated Azogado stream, in samples located in the proximity of Almadén.

The authors Esbrí and Higueras (2005) and García-Ordiales et al. (2016c) described the evolution of DHg and particulate-bound Hg (PHg) concentrations in the water from the Valdeazogues River, with background values of 30 ng L^−1^ prior to El Entredicho, maximum values in the open pit of El Entredicho around 700 ng L^−1^ and a gradual decrease in the total Hg content until the river mouth, with a value of 80 ng L^−1^. The points of the drainage network with an extremely high content of total Hg were found in the stream draining the Almadén mining area, with values that reached 2000 ng L^−1^. The proportion of DHg, more available for uptake by local fauna, was 15–45%, depending on the season and the rainfall regime.

On the other hand, García-Ordiales (2014) also analysed PTEs in the waters from the basin. Pb showed a low variability with concentrations between 0.16 and 1.00 µg L^−1^, conditioned by its low water solubility; Zn, with higher environmental availability, ranges between 3.24 and 13.00 µg L^−1^, with its maximum located in a sample taken very near Almadén, and possibly produced by human waste unrelated to mining; Cu and As variability is also quite low, varying between 0.12 and 0.53 µg L^−1^ respectively, upstream from the El Entredicho mine area and 2.70 and 3.72 µg g^−1^ also downstream, with values next to 1.00 µg g^−1^ for the majority of the river course. Total and dissolved Hg are lower than the detection limit upstream from the El Entredicho mine, area and THg reaches higher concentrations (> 1 µg L^−1^) in the lower course of the Valdeazogues basin, reaching maximum concentrations of 1.62 µg L^−1^, downstream from the confluence with the Azogado stream. The average percentage of DHg is 31.54%, and ranges between 14.81 and 40.00%. The values offered here correspond to the averages of nine samples per site (5 or 6 at some of the sites, due to the absence of water), collected between May 2010 and June 2013, and analysed by means of atomic absorption spectrometry using a Leco AMA 254 device.

## Materials and methods

### Sampling and sample preparation

Largemouth bass specimens were collected using conventional sport fishing with rod and reel angling techniques at seven locations over a 34 km stretch along the Valdeazogues River (Fig. 1). Ten specimens were obtained from the El Entredicho open pit (site PEE, Fig. 1), and were the specimens with higher variability in size and weight (Table 1). Four specimens were collected from the Castilseras reservoir (site PEC, Fig. 1), located in the middle of the transect and corresponding to a reservoir for irrigation. Another two were obtained in the La Colada reservoir (site PLC, Fig. 1); and the rest of up to 28 specimens were captured in stationary ponds (‘*tablas’*) of the river’s main course. Fish were measured (total, fork and standard length) and weighed and then kept in a cooler during the day and frozen within 6 h of collection. These fish were later thawed and dissected with a stainless-steel clinical lancet to obtain muscle samples (both hypaxial and epaxial parts) for chemical analysis. An aliquot of each sample (10 g) was also submitted to desiccation through freeze drying, using a Telstar Cryodos device; these dried samples were crushed in a KINEMATICA Microtron MB 800 B blade mill.

**Table 1:** Statistics of the main parameters measured/determined in the fish specimens

Site/N	Weight (g) ± SD	Length (cm) ± SD	THg (ng g^−1^ ww)	THg (ng g^−1^ dw)	%MeHg	Se (mg kg^−1^)	As (mg kg^−1^)	Pb (mg kg^−1^)	Zn (mg kg^−1^)	Sb (mg kg^−1^)	Cu (mg kg^−1^)	K index	Molar Ratio Se/Hg	HBV_Se_ Index
PEE/10	225.3 ± 191.0	21.5 ± 9.3	5,176.5 ± 1,158.8	18,987.0 ± 4,224.8	85.8 ± 2.1	6.9 ± 3.1	4.0 ± 1.5	12.4 ± 2.4	134.5 ± 22.3	11.3 ± 4.1	16.3 ± 4.0	1.7 ± 0.6	0.92 ± 0.2	-17.8 ± 48.7
PTH/4	166.0 ± 63.2	23.2 ± 2.8	1,510.7 ± 396.6	6,309.7 ± 2,349.0	86.2 ± 1.3	3.4 ± 0.4	2.7 ± 1.2	15.0 ± 1.1	156.4 ± 25.1	12.4 ± 2.5	18.8 ± 3.7	1.3 ± 0.1	1.37 ± 0.8	21.7 ± 4.9
PPA/4	182.0 ± 62.7	23.7 ± 2.6	1,252.2 ± 277.5	4,390.5 ± 759.6	82.4 ± 1.9	2.7 ± 0.1	3.4 ± 0.5	13.1 ± 1.2	132.8 ± 8.4	11.3 ± 2.5	14.5 ± 1.2	1.3 ± 0.1	1.56 ± 0.8	18.7 ± 4.1
PLC/2	153.0 ± 12.7	23.7 ± 0.3	1,066.5 ± 403.7	4,742.0 ± 1,207.7	85.8 ± 1.5	2.4 ± 0.1	2.5 ± 0.6	13.8 ± 0.1	146.6 ± 35.9	10.9 ± 3.1	15.4 ± 5.5	1.1 ± 0.1	1.29 ± 1.1	18.0 ± 0.1
PEC/4	106.2 ± 15.0	20.4 ± 0.8	552.0 ± 62.6	1,956.0 ± 271.7	84.5 ± 3.4	2.5 ± 0.1	3.6 ± 0.5	14.2 ± 1.5	175.6 ± 22.1	10.4 ± 2.3	12.2 ± 3.7	1.2 ± 0.1	3.25 ± 0.1	28.1 ± 0.1
PLP/2	312.0 ± 57.9	26.5 ± 1.2	1,066.7 ± 136.2	3,914.0 ± 400.2	90.6 ± 2.1	2.9 ± 0.2	2.8 ± 0.8	13.8 ± 0.5	179.4 ± 23.5	10.3 ± 0.6	17.2 ± 1.7	1.6 ± 0.1	1.88 ± 0.3	26.6 ± 5.0
PPM/2	214.0 ± 33.9	24.7 ± 1.1	994.1 ± 87.5	2,904.5 ± 30.4	90.6 ± 1.0	2.7 ± 0.1	2.9 ± 0.2	13.7 ± 1.0	158.2 ± 16.4	13.1 ± 2.9	< *DL*	1.4 ± 0.1	2.36 ± 1.7	27.9 ± 0.1

SD: standard deviation (SD not expressed when there is only one value over the detection limit); ww: wet weight; dw: dry weight; DL: detection limit. The values correspond to averages for the N specimens

In addition, at each of the sampling points, a sediment sample was taken using a Van Veen dredger from a boat. Once in the laboratory, the sediments were dried at a temperature of 25º, disaggregated, homogenized and milled in agate mortar for 2 min to obtain a grain size < 100μm fraction.

Muscle tissue was chosen for analysis in this study due to its biological relevance and its significant capacity to accumulate Hg, including MeHg, making it an important target tissue. Muscle tissue is commonly used in bioaccumulation studies because of its high protein content and its ability to reflect chronic exposure to mercury through the fish diet (Guthrie & Davis, 1977; Huckabee et al., 1979). Additionally, muscle tissue is easily accessible for sampling and provides crucial information about the body burden of Hg and MeHg in fish, which is essential for assessing potential health risks to the local population that may consume this type of fish.

To test the homogeneity of Hg distribution in the fish muscle, one specimen was divided longitudinally into three segments: close to the head, the centre and close to the tail; three muscle samples were obtained from each segment as previously described and analysed separately.

### Analyses

The analysis of the samples included the following determinations:

- Analysis of THg of wet and freeze-dried fish samples as well as of sediment samples was performed by means of Atomic Absorption Spectrometry (AAS) with a Zeeman background correction, which provides both high sensitivity and minimal interference (Sholupov & Ganeyev, 1995; Sholupov et al., 2004). The Pyro-915 + unit pyrolyses the sample and the AAS quantifies the released Hg vapour with range detection limit for fish is 1–5 µg/kg. Calibration was carried out at the beginning and the end of each analytical process, and tuna fish ERM CE 464 (THg: 5.24 mg kg^−1^) certified reference material was also analysed, showing recovery percentages of 89 and 106%. The QC/QA for sediments was based on the use of the NIST 2710A certified reference material (Montana soil), with recovery rates between 90 and 110%.
- The analysis of MeHg was performed on dry samples following the procedure described by Romero-Romero et al. (2022): each sample was digested in 5 mL aliquots of trace metal grade 4N HNO_3_ at ± 60 ◦C for 12 h (Hammerschmidt & Fitzgerald, 2006). A small volume (< 0.2 mL) of digest was added to 30 mL of deionised water, and the acid was neutralised with 8N KOH. The MeHg content was determined by purge and trap (Tenax) gas chromatographic CVAFS (Tekran 2700 analyser, detection limit 0.002 ng/L^−1^) following pH adjustment with 2 M acetate buffer and ethylation with 1% sodium tetraethyl borate (Hammerschmidt & Fitzgerald, 2001). Determinations of MeHg were performed after calibration with Brooks Rand aqueous standard solution. Duplicates and blanks were analysed for QC/QA with each sample set and DORM-2 was used as certified reference material to control the extraction procedure. The recovery percentage of MeHg in the reference material ranged between 93.4 and 96.7%. The relative standard deviations of sample duplicates were < 9% (Table TS1 in Supplementary material). DORM-2 (4.64 ± 0.26 μg g^−1^) certified reference material was used to assess the quality of analysis; the recovery percentage of MeHg in the reference material ranged between 93.5 and 96.8%, and the relative standard deviations (%RPD) of the measures were < 10%. Analysis of other elements present in the dry samples was carried out by means of X-Ray Fluorescence Spectrometry, using a MalvernPanalytical Epsilon 1 device. Samples were submitted to 21 min. of X-Ray irradiation, to obtain the fluorescence spectra, a technique which has been shown to offer satisfactory levels of precision (Higueras et al., 2017, among others). The elements considered here were Pb, Zn, Cu, As, Sb and Se. FluXana (FLX-C3) reference material was used for QC/QA, obtaining recoveries between 82 and 121%.
- Each of the analysed samples underwent several determinations to assess data variability and quality. This approach allowed us not only to evaluate result consistency, but also provide a measure of data dispersion. For the determination of total mercury (THg), we conducted three analytical determinations per sample using Atomic Absorption Spectrometry (AAS). For methylmercury (MeHg) and X-ray Fluorescence Spectrometry (FRX), two determinations were performed per sample using purge and trap (Tenax) gas chromatographic CVAFS and FRX, respectively. The Table TS1 provided reflects the average of these analyses.

### Fulton's condition factor (K)

The length–weight relationship and condition factor (K) is a useful descriptor in population fish biology, because it provides essential information on growth strategies, nutritional status, and reproduction. Also, this parameter widely used to compare the condition of aquatic systems. under varying environmental conditions or ecological factors. The study published by Cizdziel et al. (2002) describes this ‘Fulton's condition factor’ (K) as an index related to nutritional status defined by the following equation:

1 $$ {\text{Fulton}}^{\prime}{\text{s }}\;{\text{condition}}\;{\text{factor}}\;{\text{(K) = }}\left[ {\frac{{{\text{Body }}\;{\text{mass }}}}{{{\text{(Body }}\;{\text{length )}}^{{3}} }}} \right]{*100} $$

Values of this parameter > 1.0 are indicatives of a good nutritional state of the fish (Cizdziel et al., 2002).

### Selenium health benefit value (HBV_Se_)

The Se/Hg molar ratio can be used to estimate the protective effect of Se against the toxicity of MeHg (Kaneko & Ralsron, 2007). Ratios > 1 imply that the quantity of Se in the fish is enough to protect humans who consume fish from the toxic effects of MeHg (Kaneko & Ralsron, 2007; Looi et al., 2016). Additionally, Ralston et al. (2016) improves the HBV_Se_ parameter (Selenium health benefit value) proposed by Kaneko & Ralsron (2007), and expressed by the follow equation:

2 $$ {\text{HBV}}_{{{\text{Se}}}} { = }\left[ {\frac{{{\text{(Se {-} Hg)}}}}{{{\text{Se}}}}} \right]{\text{*(Se + Hg)}} $$

This parameter is used as an indicator aimed to assess human exposure to MeHg and dietary Se intake, particularly regarding maternal consumption during pregnancy (Kaneko and Ralsron (2007).

### Numerical analyses and mapping

The analytical dataset was compiled in MS Excel and processed with the Minitab 19.1 and STATGRAPHICS Centurion V.19.1.2 statistical packages. The THg distribution along the Valdeazogues River transect was obtained by inverse distance weighing using Surfer 21.1.158 (Golden Software) and ArcMAP 10.8.1 (ArcGIS) under license from UCLM.

## Results and discussion

### Specimen characteristics

The captured specimens showed some variability in size (length and weight), as shown in Table 1 for averages per site, and in Table TS1 for each of the individuals. Three specimens were clearly of a small size than the others, weighing < 20 g; 18 ranged between 80 and 250 g; and 6 ranged between 250 and 522 g. The ten specimens from the El Entredicho pit lake showed the maximum variability, including the three smallest specimens, and three of the biggest. The index related to fish nutritional status (K) for our fish are shown in Tables 1 and TS1 and are all indicative of a good nutritional status of the fish.

### Total- and methylmercury contents

Total mercury concentrations in the fish showed no significant differences along the fish itself, with 1.562 ± 11.4 ng g^−1^ ww in the head muscle (epaxial myomeres), 1.660 ± 5.3 ng g^−1^ ww in the trunk muscle (myomeres, both hypaxial and epaxial parts) and 1.530 ± 7.9 ng g^−1^ ww in the tail muscle (myosepta, both hypaxial and epaxial parts) in three individuals. In any case, and after these results, a systematic sampling of the central portion of the fish was performed wherein the values were found to be higher. Moreover, total mercury results in wet and freeze-dried samples appear to be highly correlated (r = 0.956; *p* < 0.01) Fig. S1).

On the other hand, the variability of concentrations in the different individuals is very high along the transect, with single values ranging between 473 and 7613 ng g^−1^ with an average of 2546 ng g^−1^ for wet samples, and between 1656 and 24,500 ng g^−1^, with an average of 9415 ng g^−1^ for dry samples. The comparison of these values with those published for worldwide locations (with the majority being localities from North America, where this species is native), shown in Table 2, shows that values described in this study are much higher compared to the rest: only discrete values corresponding to fish from South Carolina reservoirs in USA (Peles et al., 2006), from the Everglades National Park in USA (Julian & Gu, 2015) and from farmed fish in the Korean peninsula (Kim et al., 2012) are higher than 1000 ng g^−1^. All the measured values exceeded the maximum permissible limit issued of 500 ng g^−1^ by the WHO (THg, wet weight) (WHO, 1990), as well as the 300 ng g^−1^ US-EPA Water Quality Criterium (MeHg, wet weight) for fish muscle (USEPA, 2001).

**Table 2:** A selection of published concentrations of total Hg and methylmercury in largemouth bass

References	*N*	Unit	THg muscle (wet weight)	MeHg (%)	Study site
This study	28	ng g^−1^	489–6334	> 80.5	Almadén mining district, Spain
Davis et al. (2008)	406	ng g^−1^	530.0	ND	Sacramento River delta, San Joaquín, CA, USA
Kim et al. (2012)	196	ng g^−1^	195.0 ± 264.0 (6.9–1230.0)	ND	Farmed fishes, Korea
Knott et al. (2022)	71	ng g^−1^	299.0 (117.0–470.0)	ND	Dairy Farm Lake, MO, USA
Dharampal and Findlay (2017)	10	ng g^−1^	190.0 ± 20.0	ND	BlackWarrior River, Demopolis Reservoir, AL, USA
	10	ng g^−1^	870.0 ± 70.0	ND	Sipsey river, AL, USA
Razavi et al. (2020)	11	ng g^−1^	464.0 ± 205.0 (173.0–810.0)	> 90	Canandaigua lake, Central NY, USA
	14	ng g^−1^	201.0 ± 270.0 (21.0–881.0)		Cayuga lake, Central NY, USA
	12	ng g^−1^	302.0 ± 135.0 (161.0–535.0)		Honeoye lake, Central NY, USA
	9	ng g^−1^	51.0 ± 20.0 (26.0–95.0)		Owasco lake, Central NY, USA
	10	ng g^−1^	297.0 ± 183.0 (56.0–601.0)		Seneca lake, Central NY, USA
Gehringer et al. (2013)	11	ng g^−1^	577.0 ± 81.0 (289.0–974.0)	80	Sacramento–San Joaquín river, CA, USA
Burger et al. (2001)	48	ng g^−1^	460.0 ± 40.0	*ND*	Savannah River, SC and GA, USA
Campbell et al. (2003)	8	ng g^−1^	52.0 ± 34.9 (4.3–94.9)	*ND*	Lake Naivasha, Central Kenia
Rose et al. (1999)	153	ng g^−1^	394.0 ± 165.0	*ND*	Lakes Massachusetts, MA, USA
Julian and Gu (2015)	1136	ng g^−1^	(640.0–1820.0)	*ND*	Everglades National Park, South FL, USA
Goodchild and Gerstenberger (2011)	19	ng g^−1^	160.0 ± 700.0 (50.0–320.0)	*ND*	Ash Meadows National Wildlife Refuge, Nye County, NV, USA
Knott et al. (2019)	228	ng g^−1^	271.0 ± 129.7 (70.8–542.4)	*ND*	Ecoregion (Glacial Plains / Ozark Highlands), MO, USA
Southworth et al. (1994)	8	ng g^−1^	930.0 ± 90.0	*ND*	Lambert Quarry, Anderson County, TN USA
Paller et al. (2007)	5–19	ng g^−1^	550.0	*ND*	Savannah River, SC and GA, USA
Fernández-Trujillo et al. (2021)	6	ng g^−1^	84.0 ± 14.4 (dry weight)	*ND*	Tablas de Daimiel National Park, Ciudad Real, Spain
Peles et al. (2006)	30	ng g^−1^	690.0 ± 60.0 (190.0–1400.0)	*ND*	L-Lake, SC, USA
	26	ng g^−1^	280.0 ± 30.0 (40.0–570.0)	*ND*	Lake Marion, SC, USA
	32	ng g^−1^	300.0 ± 30.0 (30.0–810.0)	*ND*	Lake Russell, SC, USA
	21	ng g^−1^	250.0 ± 30.0 (120.0–540.0)	*ND*	Lake Thurmond, SC, USA
	30	ng g^−1^	1130.0 ± 70.0 (560.0–2010.0)	*ND*	Par Pond, SC, USA

Values expressed as average ± SD THg, and/or with range, according to the published forms; MeHg is expressed as a percentage of THg; N: number of specimens. ND: not determined

Additionally, Rimondi et al. (2012), Miklavčič et al. (2013), and Gray et al. (2014) report THg and MeHg concentrations in freshwater fish species different from *Micropterus salmoides* from the fluvial basins affected by the Idrija (Slovenia) and Monte Amiata (Tuscany, Italy) Hg mining districts. Data from Miklavčič et al. (2013) correspond to forty-four fish specimens of eight different species from the Idrija area, where concentrations for THg ranged between 92 and 1450 ng g^−1^ ww. Data from Rimondi et al. (2012) correspond to 86 fish from four species from the Paglia River, the Pagliola Creek and from a small lake located next to the mine area, with THg ranging from 160 to 1200 ng g^−1^ ww, averaging 840 ng g^−1^ ww. Gray et al. (2014) reports analyses corresponding to 54 specimens from three species from the Paglia and Tiber Rivers downstream of the Hg mining area, thus further with respect to the source area than those from Rimondi et al. (2012); the values found range from 52 to 560 ng g^−1^ ww, with a mean of 170 ng g^−1^ ww. Furthermore, Yokoyama (2018) reported for Minamata Bay that THg concentrations in undifferentiated sea fish and shellfish from the 1950s to the 1960s reached 2500–3000 ng g^−1^ ww.

**Fig. 2:**
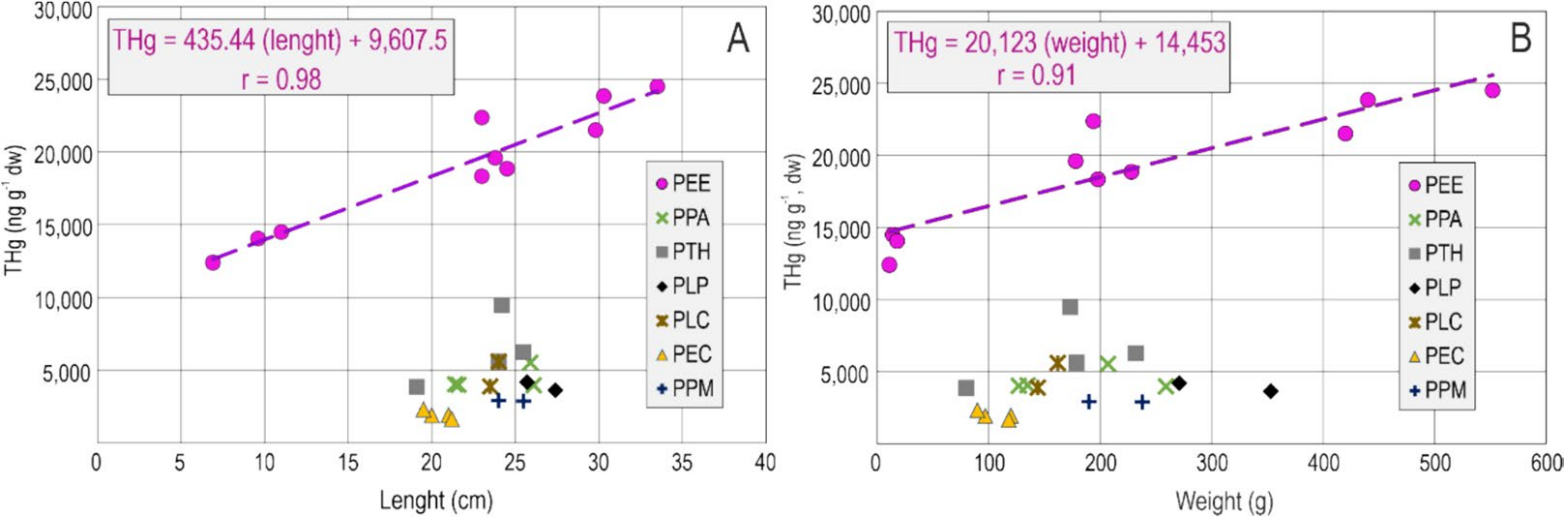
**A** Plot of THg (in ng g^−1^, dw) *vs*. length (in cm); **B** Plot of THg (in ng g^−1^, dw) *vs*. weight (in gr). Both, for the specimens captured at the seven fishing sites, with indication of the correlation parameters for the PEE site

The relationship between the THg concentration and the size-related parameters (length in centimetres and weight in grams) is expressed in Fig. 2. These correlations are specific to each sampling location as they are dependent on the available DHg in the water. The best correlation was found in the sampling site with the highest Hg content in the water (El Entredicho pit lake), and the greatest variety of specimens captured in terms of weight and length. Pearson correlations were r = 0.98 for THg (dw) *vs*. length (Fig. 2A), and r = 0.91 for THg (dw) vs. weight (Fig. 2B).

Methylmercury represents between 79.7 and 92.1% of the THg concentrations with an average of 85.9%, which is slightly lower than the 90–100% postulated by Fitzgerald and Clarkson (1991). On the other hand, MeHg reported by Miklavčič et al. (2013) represents percentages of THg in the range among 33 and 100% for the different species and sites; however, there are no clear reasons for this, since the values < 90% are from different sites and a variety of fish species.

### Other PTEs

As shown in Table 1, the concentrations of other PTEs display moderate variability with averages and standard deviations (in mg kg^−1^ dw) of 13.3/1.8 for Pb; 149.1/26.1 for Zn; 15.4/3.9 for Cu, 3.4/1.1 for As; 11.3/2.9 for Sb; and 4.9/3.1 for Se. These results are comparable with data provided by Fernández-Trujillo et al. (2021) in *Micropterus salmoides* from “Tablas de Daimiel” National Park, located some 105 km to the NE of Almadén: 200 ± 58 mg kg^−1^ for Pb; 111 ± 39 mg kg^−1^ for As; and 4.3 ± 1.65 mg kg^−1^ for Se, higher than in our study for Pb and As but similar for Se.

**Fig. 3:**
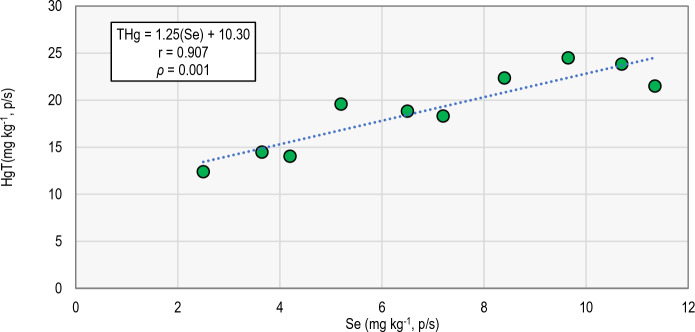
Plot of THg *vs*. Se in the El Entredicho pit lake, with the indication of the correlation parameters

The concentrations of these elements are not conspicuously correlated to that of Hg, except for Se (Fig. 3), which indicates differences in the absorption patterns of these elements. As indicated in the introduction, this relationship between Hg and Se appears to be a constant in many organisms, and the common increase in both elements seems to be a consequence of the protective character of Se against the toxicity of Hg. On the other hand. Fernández-Trujillo et al. (2021) found similar Se concentrations in this (and other) fish species in a pristine context (almost free of Hg), although with high concentrations of other PTEs. These authors indicate that this ‘anomaly’ of Se concentration in fish can be a consequence of the drainage of Se-rich soils (Locutura-Rupérez et al., 2012) by the fluviatile system feeding this wetland, causing high Se contents in the sediments of the area (Jiménez-Ballesta et al., 2017).

The study by Lemly et al. (1996) indicates that Se concentrations in the range of 4000—8000 ng g^−1^ dw can produce blood changes, reduced growth, mortality in juveniles and reproductive failure in several fish species. However, as previously indicated, the K index (Table 1, and TS1 for all the exemplars) does not provide any indication of negative health effects on any of the studied specimens, even in the extreme conditions of the El Entredicho pit lake.

**Fig. 4:**
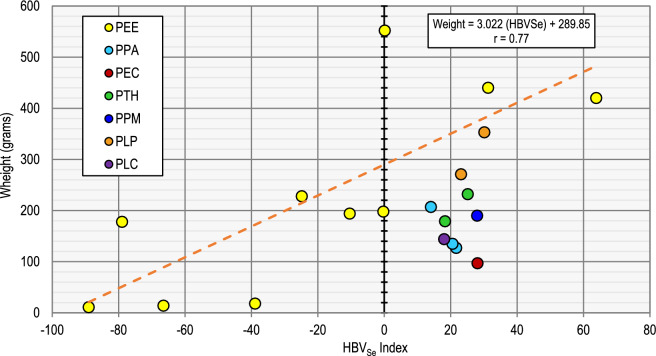
Plot of weight of fishes (in grams) *vs*. the HBV_Se_ index, with the indication of the correlation parameters for PEE

As shown in Table 1, as well as in Table TS1 for the individuals, most of the studied exemplars have ratios > 1; only those from the El Entredicho pit lake (site PEE) do not exceed this value. On the other hand, higher ratios were found in individuals from the Castilseras and La Pedrona reservoirs (sites PEC and PLP), which suggests that these sites, also characterised by the lowest THg and MeHg concentrations in fish, have the lowest risks for fishing and using the captured exemplars as food. Results for this parameter, based on Eq. 2, demonstrate than most of the analysed individuals show positive HBV_Se_ values, including those from the El Entredicho pit lake with a weight > 300 g (Fig. 4). But most specimens with a weight < 300 g from this site show negative values, demonstrating that these, likely the youngest individuals, represent a higher risk for consumption than the larger, probably older ones. The use of this fish as food is forbidden locally, minimising the risks to the human food chain.

### Geographic distribution of concentrations

**Fig. 5:**
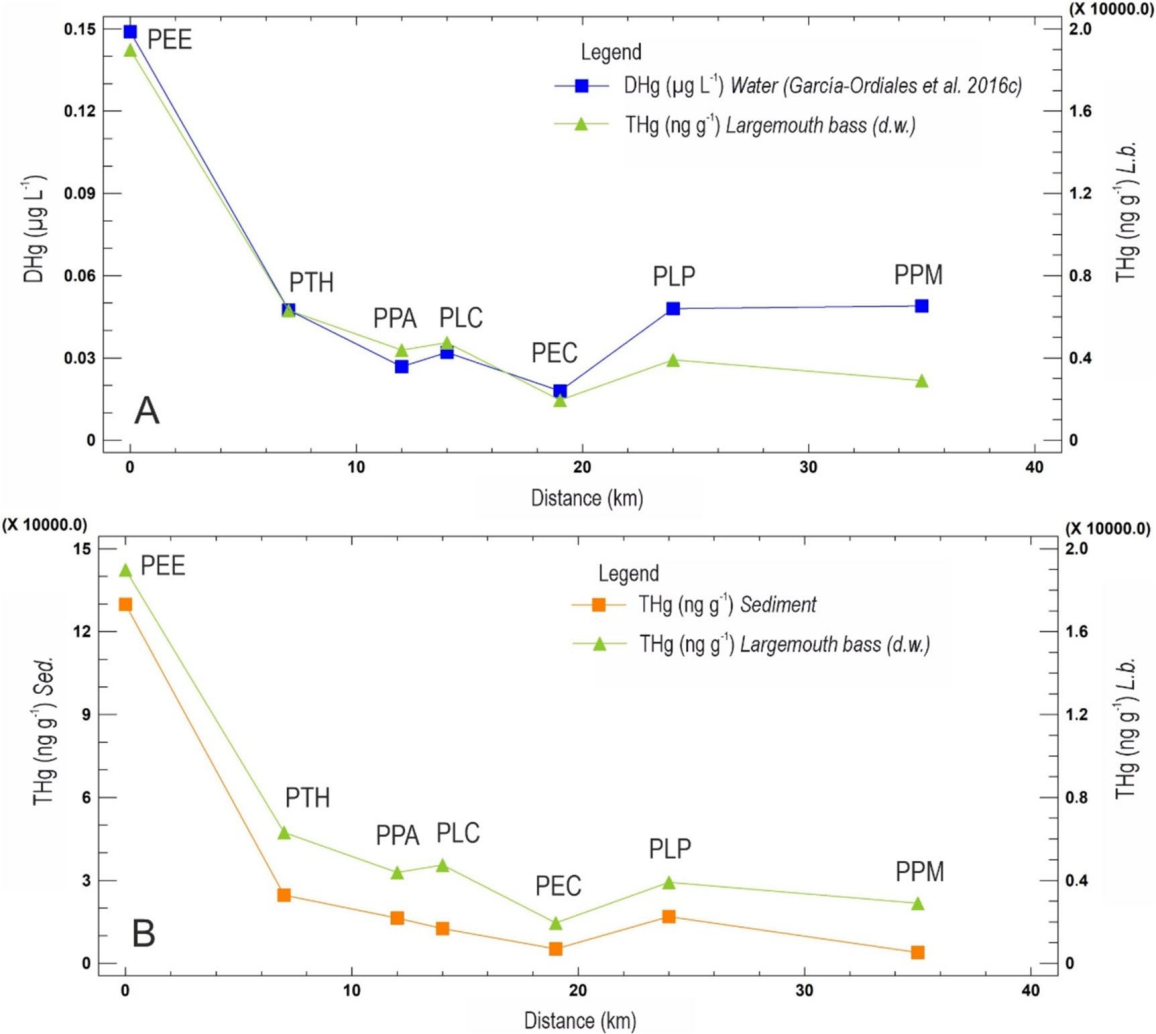
**A** Plot of THg concentrations in fish and water in the transect. The starting point for distances along the Valdeazogues River is the El Entredicho open pit (site PEE). **B** Plot of THg concentrations in fish and sediment in the transect. Concentrations in the water correspond to those from García-Ordiales et al. (2016c)

The concentrations of Hg in fish along the studied transect (Table 1 and Fig. 5) show a clear trend with a very high maximum in the El Entredicho pit lake (PEE, Figs. 1 and 5), and a progressive decrease downstream. As an anomaly in this trend, the Castilseras reservoir site (PEC, Figs. 1 and 5) shows the minimum values, with four exemplars with THg in the range 473–605 ng g^−1^ dw. Water from this reservoir showed high concentrations of THg in 2003 (Gray et al., 2004) as well as DHg and PHg concentration being 32 and 15 ng L^−1^ respectively in 2016 (García-Ordiales et al., 2016c). On the other hand, the Castilseras reservoir corresponds to a water mass with a larger extension and water column. In this context, the dynamics of input water and the input of clean water by immediate tributaries may facilitate the continuous cleaning of the reservoir’s water, reducing the presence of DHg and therefore also reducing the THg bioaccumulation in the biota. This process should be the opposite of what occurs in the El Entredicho pit lake, as there is no possibility for retained water to flow downstream, and thus acts as a closed system. This is the most important difference between both sites and could explain the anomalously low concentrations of THg in fish at the Castilseras reservoir area. It is necessary to mention that the only important and active source of DHg and PHg emissions is located in El Entredicho pit lake, which was disconnected from the Rio Valdeazogues in 2004, so the dynamics of this isolated lake are completely different from that of the Castilseras reservoir, which is renewed by multiple tributaries with no known Hg emission sources.

As also displayed in Fig. 5, the concentrations of THg in fish show a highly coincidental declining trend with concentrations in water (data from García-Ordiales, 2016c) and sediments. This trend is not likely to have an influence on the presence/absence of these fish species since they do not show associated toxicity and migration along the river occurs preferentially due to dry periods and the formation of water ponds that persist with water during the summer. In the same way, Fig. 5 plots the concentrations of THg in fish and in sediments, considering the points of coincidence between the fishing sites and the sampling points. The pattern of mercury bioaccumulation in fish muscle appears to be influenced by several factors related to the bioaccumulation and biomagnification of mercury within the aquatic ecosystem.

Even though the studied specimens primarily absorb Hg from the DHg in the water column, sediments have also been shown to be an important source of Hg transfer between them and the water column (Bloom et al., 2003; Caplat et al., 2005; García-Ordiales et al., 2020).

The comparison of the graphs shown in Fig. 5A, B reveals that both DHg in the water column (data from García-Ordiales et al., 2016c) and THg in the sediments exhibit similar distribution patterns along the river and are associated with mercury concentrations in the fish. The high concentration in the initial DHg and THg sample at the PEE, location is related to the mining activities at the Mina del Entredicho, which subsequently decreases downstream, possibly due to the input of non-impacted materials and waters.

In the final stretch of the river, there is a noticeable increase in mercury concentrations. This rise is attributed to the contributions from the Azogado creek, a well-known source of mercury contamination in the district's waterways (Gray et al., 2004 and Higueras et al., 2006).

For the rest of the PTEs, and considering only fish vs. sediments, we have not found any relationship amongst their concentrations for the different sites (Table TS1).

## Conclusions

Our study, based on a 34 km long survey in one of the most Hg-contaminated rivers worldwide, has offered the following conclusions:

- The studied fish (*Micropterus salmoides*) have a very high Hg bioaccumulation capacity. The average general Hg concentration in their muscle is 2545.7 ng g^−1^ for wet samples (range: 473–7613 ng g^−1^), and 9415 ng g^−1^ for dry samples (range: 1656 and 24,500 ng g^−1^). These concentrations are much higher than the maximum allowed level for fish consumption of 500 ng g^−1^ dw. Only one specimen (among the 28 studied) showed concentrations lower than this threshold.
- Methylmercury concentrations measured in the survey confirmed the previous data on this fish species: it usually represents a very high percentage of THg, in this case, in the range of 79.7–92.1%.
- The concentrations of THg and MeHg measured in this transect, and in particular in the El Entredicho pit lake, are the highest ever recorded not only in this species, but in all the published literature on this matter. For instance, fish from the Minamata Bay contained THg concentrations in the range of 2500–3000 ng g^−1^. These data suggest that the ban on the use of these fish as food should be maintained.
- Other PTEs analysed in the studied specimens show values of low significance, even though the Almaden mining district is rich in decommissioned mines which exploited these elements, particularly Pb and Sb. It is possible that the low environmental mobility of these elements limits their uptake and bioaccumulation on lower chains on the food web.
- The case of Se is different. This element has been widely described as associated to Hg in organisms, acting as a protective agent against the toxicity of MeHg. As for the case of Hg, Se concentrations in the studied fish are extremely high, almost defined as toxic, particularly when Hg concentrations are also very high. These extreme concentrations in both elements do not appear to have deleterious effects on the health of the fish.
- The analysis of the Se/Hg molar ratio and/or the HBV_Se_ parameter should be an indication that the risks for human intake of *M. salmoides* is low, except for the smallest, youngest individuals.
- The geographic distribution of concentrations of THg follows a clear scheme, with the very highest values in an inundated pit lake corresponding to a historic Hg mine, located upstream from the survey area. Downstream from this site, values decrease steadily, with some sites showing lower-than-expected concentrations for this general scheme.
- Finally, it is important to highlight that although mercury mining and metallurgical activity came to a halt in the district by 2008, there remain considerable consequences in terms of the pollution of the hydric system and the related biota, and there continues to be a risk to the regional food chain.

Additional research is needed to better understand and clarify the relationship among the THg concentrations in fish muscle and the characteristics of the sites where the fish live; in this aspect, the Valdeazogues basin has proven to be a very appropriate area, with separated contexts with differentiated characteristics in terms of water volume, water column height, and concentrations and speciation of the element in water and sediments. In addition, in future research will be important to control the parameters not considered here, such as the age and sex of the individuals, food preferences by site, other fish species, variabilities in THg and MeHg concentrations and (bio)availability in and sediments, the characteristics of the water body, the depth, the food web and prey, and their THg and MeHg concentrations.

## Supplementary Information

Below is the link to the electronic supplementary material.

Supplementary file1 (JPG 1009 kb)

Supplementary file2 (JPG 773 kb)

Supplementary file3 (JPG 407 kb)

Supplementary file4 (JPG 397 kb)

Supplementary file5 (XLSX 15 kb)

## Data Availability

No datasets were generated or analysed during the current study.
