# Supplementary figures and images for: A preliminary assessment of mercury, methylmercury and other potentially toxic elements in largemouth bass (Micropterus salmoides) from the Almadén mining district
Source: Environ Geochem Health. 2024 Dec 23;47(1):27. doi: 10.1007/s10653-024-02326-3 (PMC11666705; doi:10.1007/s10653-024-02326-3)

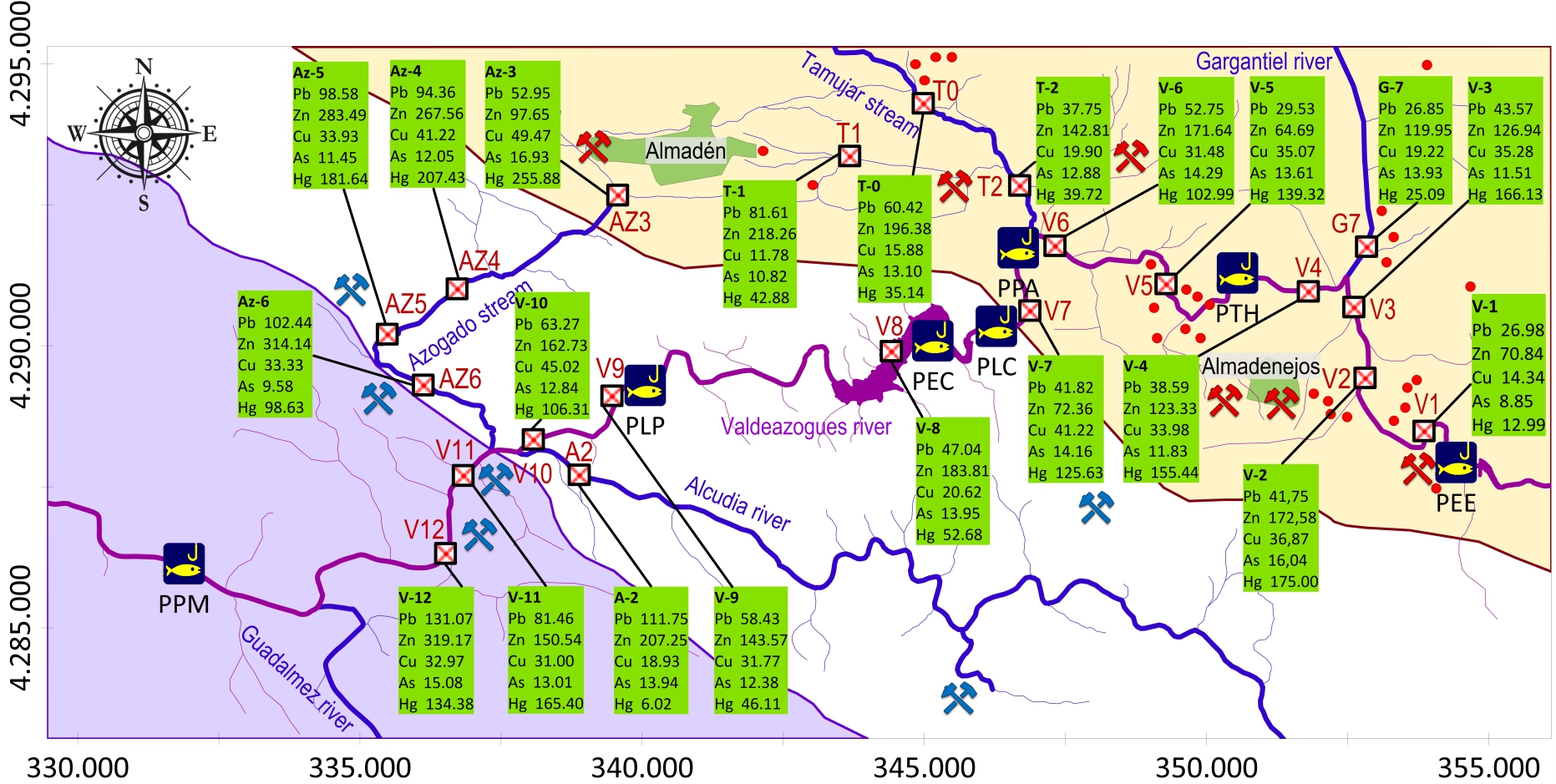

Supplement: Supplementary file 1 — Supplementary file1 (JPG 1009 kb) [file 10653_2024_2326_MOESM1_ESM.jpg]

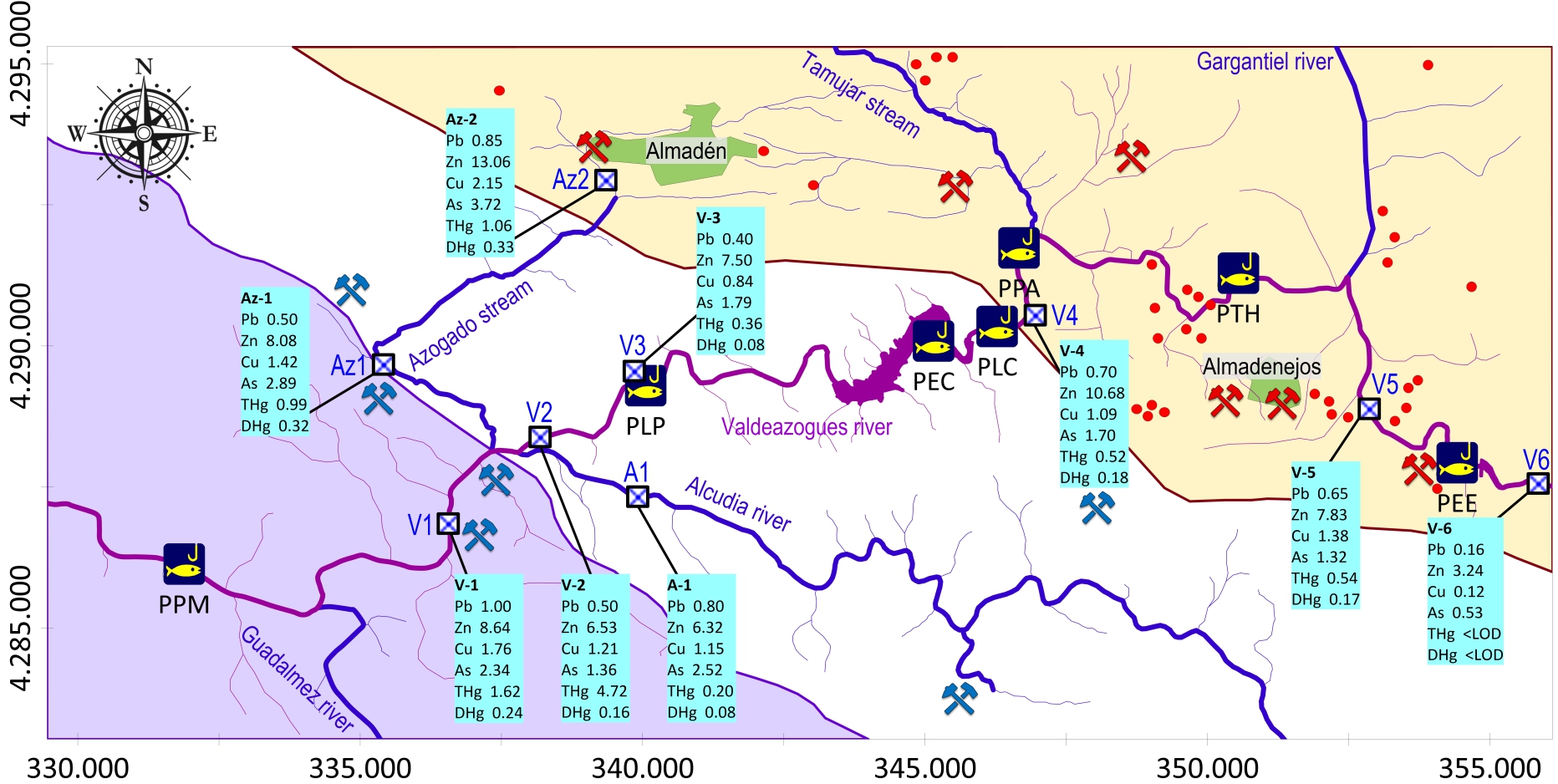

Supplement: Supplementary file 2 — Supplementary file2 (JPG 773 kb) [file 10653_2024_2326_MOESM2_ESM.jpg]

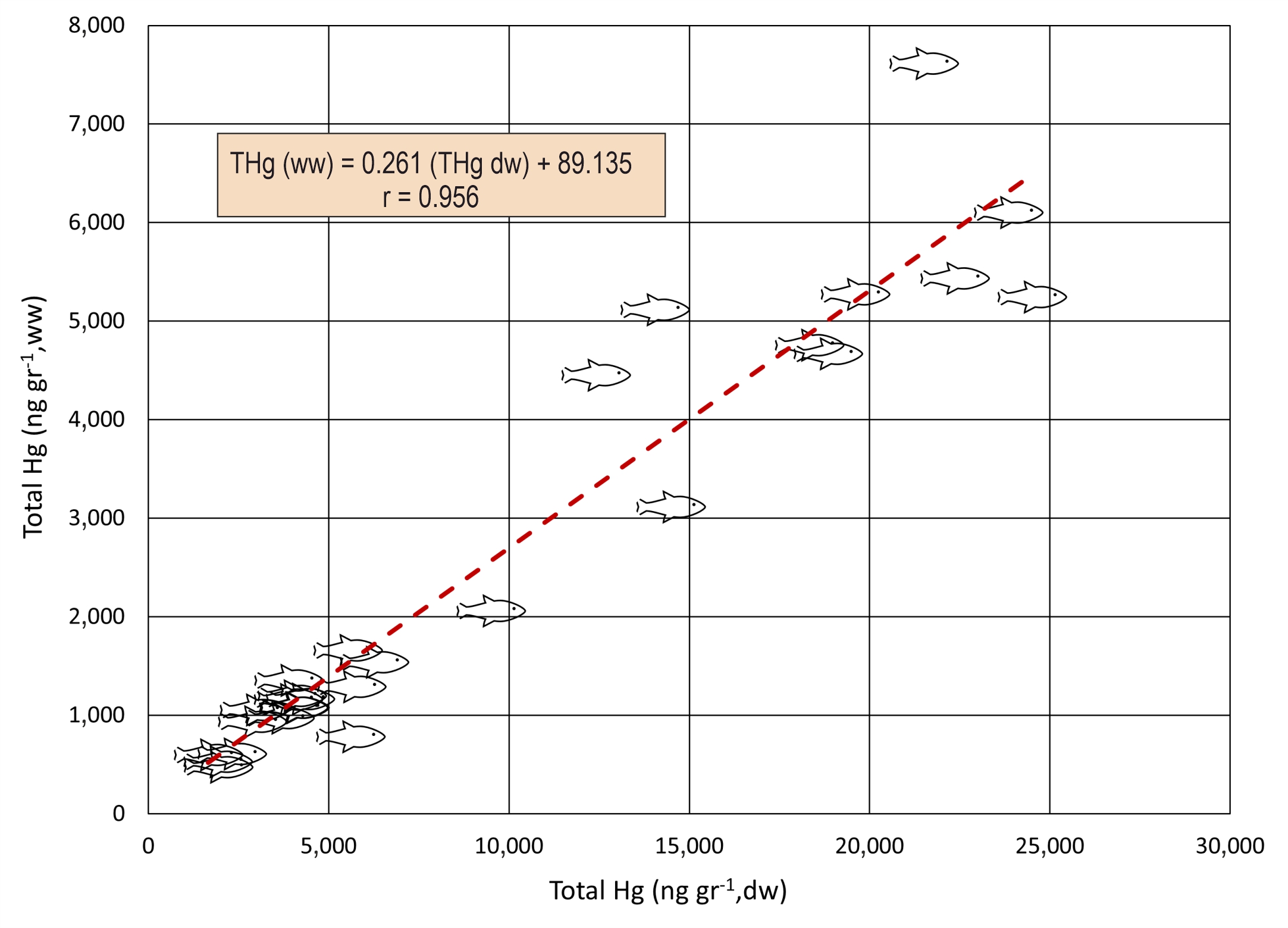

Supplement: Supplementary file 3 — Supplementary file3 (JPG 407 kb) [file 10653_2024_2326_MOESM3_ESM.jpg]

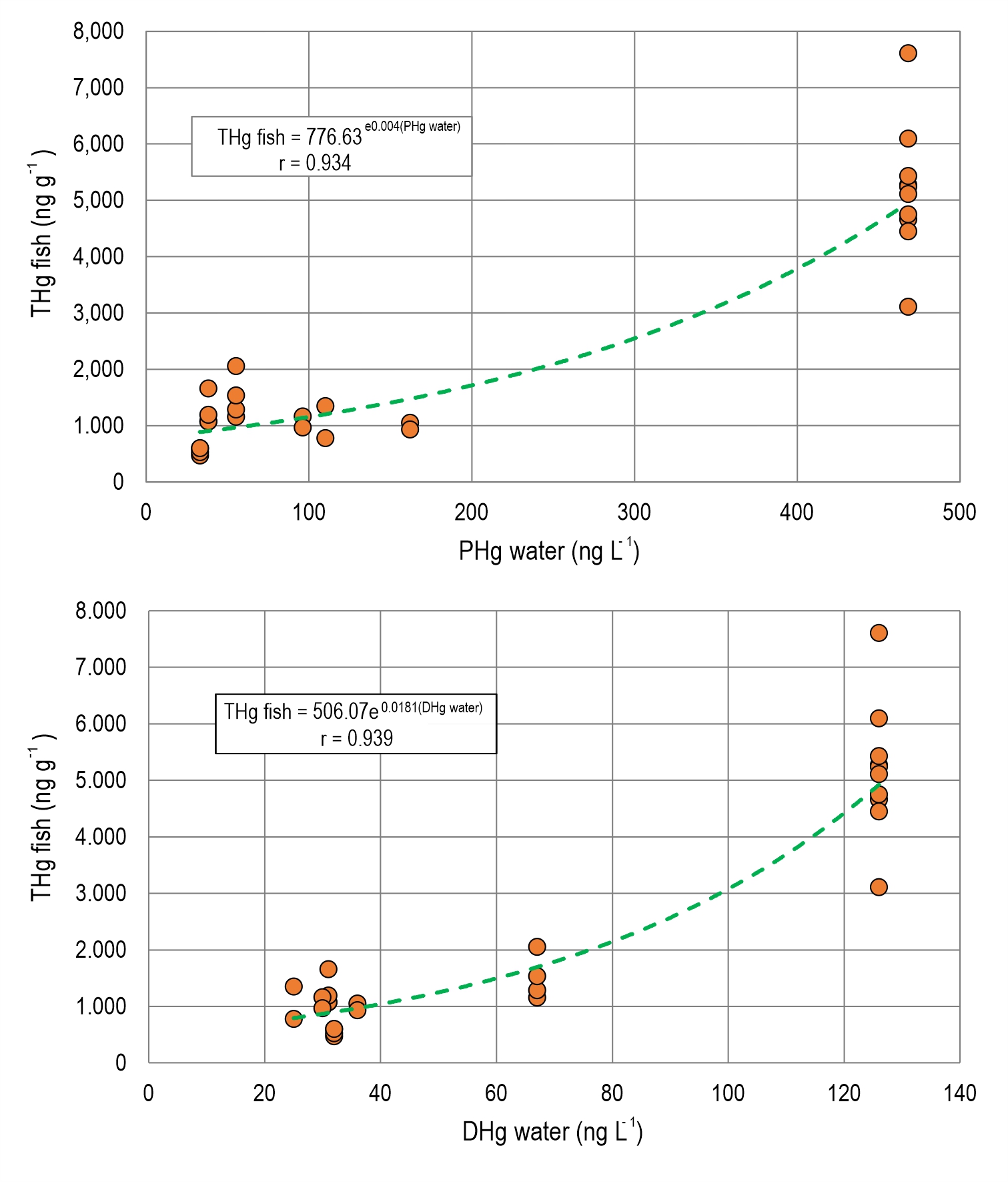

Supplement: Supplementary file 4 — Supplementary file4 (JPG 397 kb) [file 10653_2024_2326_MOESM4_ESM.jpg]
